# Aboriginal birth cohort (ABC): a prospective cohort study of early life determinants of adiposity and associated risk factors among Aboriginal people in Canada

**DOI:** 10.1186/1471-2458-13-608

**Published:** 2013-06-25

**Authors:** Gita Wahi, Julie Wilson, Ruby Miller, Rebecca Anglin, Sarah McDonald, Katherine M Morrison, Koon K Teo, Sonia S Anand

**Affiliations:** 1 McMaster University, 1280 Main Street West, Hamilton, ON L8S 4K1, Canada; 2 Six Nations Health Services, 1745 Chiefswood Rd, Ohsweken, ON N0A 1M0, Canada; 3 Population Health Research Institute, Hamilton Health Sciences and McMaster University, Hamilton, Canada; 4 Population Genomics Program, Chanchlani Research Centre, McMaster University, Hamilton, Canada

**Keywords:** Aboriginal, Birth cohort, Early origins, Adiposity

## Abstract

**Background:**

Aboriginal people living in Canada have a high prevalence of obesity, type 2 diabetes, and cardiovascular disease (CVD). To better understand the pre and postnatal influences on the development of adiposity and related cardio-metabolic factors in adult Aboriginal people, we will recruit and follow prospectively Aboriginal pregnant mothers and their children – the Aboriginal Birth Cohort (ABC) study.

**Methods/design:**

We aim to recruit 300 Aboriginal pregnant mothers and their newborns from the Six Nations Reserve, and follow them prospectively to age 3 years. Key details of environment and health including maternal nutrition, glucose tolerance, physical activity, and weight gain will be collected. At birth, cord blood and placenta samples will be collected, as well as newborn anthropometric measurements. Mothers and offspring will be followed annually with serial measurements of diet and physical activity, growth trajectory, and adiposity.

**Discussion:**

There is an urgent need to understand maternal and child factors that underlie the early development of adiposity and type 2 diabetes in Aboriginal people. The information generated from this cohort will assist the Six Nations community in developing interventions to prevent early adiposity in Aboriginal children.

## Background

People of Aboriginal ancestry are a rapidly growing population in Canada. There are over 1.2 million Aboriginal people living in Canada, and the birth rate among Aboriginal people is 1.5 times that of the general population [1]. Aboriginal adults suffer a high prevalence of obesity, type 2 diabetes, and cardiovascular disease (CVD) compared to their non-Aboriginal counterparts [2]. Furthermore, the prevalence of type 2 diabetes among Aboriginal youth is disproportionately higher compared to other youth in Canada [3]. The increased prevalence of obesity and diabetes among Aboriginal people is highly correlated with their adoption of western lifestyle practices (i.e. high energy intake and low physical activity). However, emerging evidence suggests that the propensity to develop obesity and type 2 diabetes is influenced by both the pre and postnatal environment which shapes developmental growth trajectories throughout the offsprings’ life [4].

To better understand the pre and postnatal influences on the development of adiposity and related cardio-metabolic factors (i.e. abnormal glucose, insulin, blood pressure, and lipids) in Aboriginal people, we propose to recruit and follow prospectively Aboriginal pregnant mothers and their offspring from the Six Nations Reserve near Brantford, Ontario and surrounding areas. This Aboriginal birth cohort (ABC) study will enhance our understanding of the determinants of adiposity, type 2 diabetes and related cardio-metabolic factors in Aboriginal people, with the ultimate goal of developing chronic disease prevention strategies for this high-risk group.

### Study rationale

#### Aboriginal people of the Six Nations

The Six Nations Reserve in Brant County, Ontario, Canada took its present form of 20,000 hectares in 1847, and is now home to over 12,000 Aboriginal people [5]. The traditional lifestyle of the Six Nations people included agricultural farming, hunting and fishing but the increase in permanent settlements during the second half of the 20th century led to their growing dependence on store-bought foods, and an increased dependence on automobiles and other energy-saving devices. Since 1998, our research group has worked closely with the people of the Six Nations to document CVD risk factors, and subsequently to facilitate interventions to reduce these risks for CVD [2,5-13]. In a previous study we conducted among the Six Nations adults, 60% of men and 55% of women were obese (BMI (≥30 kg/m^2^), compared to 32% and 24% of non-Aboriginal men and women in Canada respectively [2]. In addition, the prevalence of glucose intolerance, dyslipidemia, tobacco use, and CVD was significantly higher among Six Nations adults compared to age and sex matched Canadians of European origin [2]. Furthermore, community level factors such as the built environment, access and affordability of healthy foods, and easy access and affordability of tobacco likely influences the development of adverse health behaviours [14]. More recently our investigations have focused on pregnancy and early childhood as it represents a time period when chronic disease prevention may have the greatest impact [15].

#### Early origins of adiposity and related metabolic changes

Childhood obesity is attributable to environmental changes leading to increased energy intake and lower physical activity. Despite significant population-level changes, there is increasing evidence that a complex interplay of genetics, epigenetics, and non-genetic factors also interact to “program” a newborn to be more or less prone to develop excess adiposity depending on the environment to which it is exposed [16]. The focus of this study is to determine the key exposures (before, during, and after pregnancy), which strongly influence the offspring’s weight and adiposity from birth until early childhood.

#### Rationale for the creation of an Aboriginal birth cohort

There are numerous birth cohorts underway around the world, and most are being conducted among white Caucasian populations. To our knowledge, there are no Aboriginal birth cohorts in Canada and in addition to the PIMA Indian studies in the US [17] we only identified one other birth cohort study being conducted in Indigenous people in Australia [18]. Creation of an Aboriginal specific birth cohort is important for three reasons: 1) among children and youth of Canada, Aboriginal children have the highest rate of type 2 diabetes [3], and are therefore are a high risk population, 2) robust health information derived from within Aboriginal communities will provide them with culturally specific information needed to plan prevention programs [19], and 3) multi-ethnic comparisons between high risk and low risk groups lead to new understandings of disease pathogenesis as we have demonstrated among adults [11].

#### Contextual factors which influence adiposity

The Six Nations community suffers from extensive socioeconomic hardship with high rates of unemployment, low income, and only a small proportion of community members have post-secondary education [9]. This social disadvantage is strongly associated with obesity, tobacco use, diabetes and CVD among the Six Nations people [9]. Socioeconomic status (SES) also strongly influences the home environment provided to the newborn and is likely associated with health behaviours including breast feeding, dietary intake, tobacco use, and activity patterns. Low SES is also associated with maternal health post partum including mental health conditions (i.e. depression and anxiety), as well as domestic violence [20,21]. Aboriginal women in Canada are much more likely to have low household incomes, greater social disadvantage, and greater psychosocial stressors compared to non-Aboriginal women, and there is sparse data regarding social support [22]. It is likely that low SES interacts with other risk factors, i.e. diet, activity, alcohol intake, and smoking which together contribute to adverse birth outcomes and increased infant morbidity [23]. In ABC we measure key contextual factors (i.e. SES, antenatal and postpartum depression, domestic violence, and social support) and will test their association with feeding, activity and adiposity in the growing offspring.

#### Genetics and epigenetics

To our knowledge the contribution of common genetic polymorphisms to the development of adiposity and cardio-metabolic traits has not been comprehensively investigated among newborns. Furthermore, the modulation of the genetic effects by *in utero* characteristics, postnatal diet, activity, and postnatal rate of weight gain in relation to the growing offspring’s adiposity has not been investigated. There is emerging evidence from model systems and human placentas that early exposure to environmental factors – including *in utero* environment – produce epigenetic modifications leading to changes in gene expression, metabolic profile, and infant growth [24]. Maternal malnutrition, overnutrition and smoking exposure can induce epigenetic modifications of the fetal genome. The impact of early environmental exposures on epigenetic markers has not been systematically studied in humans partly due to technological barriers. However chip-based epigenetic measurements can now be made with a high degree of precision and reproducibility. Creation of ABC with its longitudinal assessment of nutritional and metabolic risk factors during pregnancy (i.e. fetal environment), at birth and up to 3 years of age for the index child, provides a unique opportunity to characterize how early environmental exposures interact with genetic variants that in turn program lifelong adverse health trajectories. By studying mothers and their offspring, and through our participation in large genetics consortia, we will be able to study gene–environment interactions on the development of risk factors and adiposity in early childhood.

### Study objectives

#### Primary objectives

1. Determine the major antenatal maternal factors (e.g. pre-pregnancy weight, weight gain, dietary intake, physical activity, and smoking exposure), selected paternal factors (e.g. cigarette smoking), and pregnancy factors (e.g. maternal weight gain, smoking exposure, glucose intolerance, and pregnancy-induced hypertension) which are associated with the newborn’s adiposity and cardio-metabolic factors at birth, and annually for the first three years of life.

2. Determine the association between early feeding practices (i.e. exclusivity of breastfeeding, formula feeding, type, frequency and duration of breast/bottle feeding, and growth after weaning), sleep patterns and activity on newborn’s adiposity, and related cardio-metabolic factors annually for the first 3 years of life.

3. Determine the impact of the home environment, including socio-economic status, social support, and maternal psychosocial factors on newborn’s adiposity at birth and annually for the first three years of life.

### *Secondary objectives*:

4. To determine if the rate of breastfeeding increases with a prenatal education and breastfeeding training focused on a family member or support person.

5. To study the association between selected genetic variants and epigenetic marks of the mother and offspring on the offspring’s adiposity and related cardio-metabolic factors.

6. To investigate the association between maternal diet in pregnancy, and infant diet with the infant microbiome at 1 year, and determine the association between the infant microbiome and child health outcomes.

7. To determine if there are differences in birth weight and adiposity (corrected for gestational age and sex) comparing Aboriginal newborns to an existing cohort of white Caucasian and South Asian newborns in Canada.

8. To qualitatively explore grandmothers’ beliefs regarding optimal health behaviours for women during the perinatal period, to determine how these compare to evidence-based knowledge about health behaviours and to identify opportunities for knowledge translation interventions.

## Design/methods

We propose to recruit 300 Aboriginal pregnant mothers and their newborns from the Six Nations Reserve and follow them prospectively to the age of 3 years. Approval was received from the McMaster/Hamilton Health Sciences Research Ethics Board (REB) on April 19, 2012 as well as the Six Nations Band Council REB on May 22, 2012.

### Inclusion criteria

Women of Aboriginal ancestry who are pregnant.

### Exclusion criteria

Women who conceived the fetus using artificial methods including in-vitro fertilization or intrauterine insemination, women carrying more than one fetus, surrogate mothers, women who suffer from severe chronic medical conditions including active cancer, severe infectious diseases including HIV, hepatitis B or C, or who are VDRL positive, will be excluded (Table 1).

**Table 1:** Eligibility criteria

**Inclusion criteria**	**Exclusion criteria**
Women < 40 years old	Women who conceived the fetus using artificial methods including in-vitro fertilization or intrauterine insemination
Aboriginal ancestry	Women carrying more than one fetus
Pregnant with singleton	Surrogate mothers
	Women who suffer from severe chronic medical conditions including active cancer, severe infectious diseases including HIV, hepatitis B or C, or who are VDRL positive

### Data collection

Stage I: Antenatal data

Pregnant mothers are recruited through self referral, or referral to the study from local health care providers (midwives, nurses, primary care physicians, obstetricians). A log of all interested subjects is kept, and the main reasons for exclusion or refusal to participate is recorded. At the baseline visit, between 24–28 weeks of pregnancy, information on age, parity, medical and pregnancy history, cigarette smoking exposure of mother, father and family members, drugs and alcohol exposure, family structure (i.e. marital status, and number of children in the house), community of birth, mother tongue, cultural practices, psychosocial characteristics, as well as socioeconomic factors (i.e. household income, education, and employment) is collected (Table 2). The pregnant mother also has a number of anthropometric measurements taken during this visit. A digital scale is used to record body weight to the nearest 100 g. Height is measured using a stadiometer to the nearest 1 cm and mid upper arm circumference to the nearest 0.1 cm using a plastic measuring tape. Maternal BMI is calculated using weight and height at baseline (kg/m^2^). The mother’s pre-pregnancy weight is recorded. Skinfold thickness (triceps and subscapular) is measured to the nearest 0.2 mm, using skinfold calipers (Holtain, UK), for the prediction of body fat using prediction equations [25]. Systolic and diastolic blood pressure is measured using an automated BP monitor (OMRON Intelli Sense, Model HEM-757). All ultrasound reports are obtained and used to establish gestational age and to assess fetal growth characteristics.

**Table 2:** Proposed measures and timing of measures in ABC

**Measures**	**Antenatal visit**^**1**^	**Birth visit**	**6 months post delivery (telephone)**	**1 year visit**	**2 year visit**	**3 year visit**
**Demographics -**	X					
Age	X			X^1^	X^1^	X^1^
HCN	X			X^1,3^	X^1,3^	X^1,3^
Address/Postal Code	X			X^1^	X^1^	X^1^
Family Doctor - Info	X			X^1^	X^1^	X^1^
Midwife/ObGyn Info	X					
Expected Delivery Date	X					
**Medical History**	X					
Diabetes	X					X^1^
Increased blood pressure	X					X^1^
Increased cholesterol	X					X^1^
Other major medical history	X					X^1^
Family History	X					
Medications Used	X					X^1^
**Past Pregnancy Info**	X					
GTPAL	X					
Still Births	X					
Past Gest. DM	X					
Pre-Eclampsia	X					
Low Birth Weight	X					
Premature Birth	X					
**Social Determinants**	X					
Years Living on the Reserve	X					
Place of Birth	X					
Religious Practices	X					
Annual Household Income	X			X^1^	X^1^	X^1^
Occupation	X			X^1^	X^1^	X^1^
Marital Status	X			X^1^	X^1^	X^1^
Education	X					X
Social Support	X			X^1^	X^1^	X^1^
Domestic Violence	X			X^1^	X^1^	X^1^
Depression	X		X^1^	X^1^	X^1^	X^1^
**Health Behaviours**						
Cigarette Exposure	X			X	X	X
Diet/Infant feeding	X		X^3^	X^3^	X^3^	X^3^
Activity/Sedentary Behaviours	X		X^3^	X^3^	X^3^	X^3^
**Physical Exam**	X			X	X	X
Blood Pressure	X	X^2^		X^3^	X^3^	X^1,3^
Height/Length	X	X^2^	X^3^	X^3^	X^3^	X^1,3^
Weight	X	X^2^	X^3^	X^1,3^	X^1,3^	X^1,3^
Waist and Hip Circumference	X	X^2^		X^3^	X^3^	X^1,3^
Skin Folds	X	X^2^		X^3^	X^3^	X^1,3^
Head Circumference (baby only)		X^2^	X	X	X	X
Fetal Ultrasound	X					
**Blood Analysis**	X					
Hemoglobin	X					
Glucose		X^2^		X^3^	X^3^	X^3^
75 g OGTT (0, 60, 120 min)	X					
Insulin	X	X^2^		X^3^	X^3^	X^3^
Adiponectin	X	X		X	X	X
Leptin	X	X^2^		X	X	X
Lipid Profile	X	X^2^		X^3^	X^3^	X^3^
CBC		X^2^		X^3^	X^3^	X^3^
Aliquots for Future Analysis	X	X^2^		X^3^	X^3^	X^3^
DNA Long-term Storage	X	X^2^				
**Birth Visit**						
Type of Delivery		X				
Duration of Labour		X				
Premature Labour		X				
Blood Loss		X				
Birth Weight		X				
APGAR scores (1 and 5 min)		X				
Adverse outcomes		X				
Placenta & Cord Blood		X				
Stool				X^3^		
Breastmilk		X^4^				

^1^: All measurements taken in the mother, ^2^: Measurements taken in newborn at birth, ^3^ Measurements taken in infant, ^4^: Collected from mother at 6 weeks.

*Dietary and Physical Activity assessment:* We have previously developed and validated a FFQ for Aboriginal people in Canada [8], which is administered during the second trimester visit, as well as at 6 months and 1 year postpartum visits. Information on maternal activity during pregnancy is collected for activities in 5 domains – occupational, discretionary exercise, household chores, sedentary activities, hobbies and sleep. Maternal sedentary behaviours will include daily screen time (computer, television, video games). Activity and sedentary behaviour are collected at baseline and at each annual visit.

*Psychosocial Assessment:* SES is assessed by recording annual household income, employment, education and marital status. Information is gathered about chronic stressors in the home, workplace and community and stressful life events. Adequacy of social support to the mother is measured using a questionnaire to evaluate the emotional, instrumental, informational, and appraisal components of social support. Depression in the mother is assessed by the Kessler-10 scale (K-10) which is a 10-item scale with five response categories ranked on a 5-point scale [26]. Intimate partner violence is assessed using the 2-item Woman Abuse Screening Tool short version [27]. All psychosocial questionnaires are administered at the baseline visit, at 6 months postpartum and annually thereafter.

*Laboratory Assessments:* The classification of maternal glycemic status is critical to determine glucose-metabolic status during the second trimester of pregnancy. All non-diabetic mothers will undergo the 75-gram oral glucose tolerance test (OGTT) between 24–28 weeks of gestation. This test is chosen to avoid the high false negative rate using the 50 gram glucose challenge test among some non-white populations [28]. Three blood samples are collected: fasting, 60, and 120 minutes [29]. Some local analysis are performed immediately (i.e. glucose, complete blood count) using standardized assays, and the remainder are processed, shipped and stored at the Clinical Trials Clinical Research Laboratory (Hamilton Health Sciences) for future analysis (i.e. lipids, adiponectin, leptin, insulin, the buffy coat for DNA extraction).

Stage 2: Birth

At the time of birth, details including birth outcomes for the mother and baby (e.g. type of delivery, APGAR scores, problems during delivery, length of stay) are collected. A cord blood sample for biochemistry (i.e. glucose, insulin, lipids, adiponectin, leptin), DNA and additional serum and plasma aliquots for future analysis is collected from each baby. Newborn’s physical characteristics including birth weight, skin fold thickness, length, abdominal, head, and arm circumference and blood pressure are measured within 72 hours after birth.

*Assessment of Body Composition in Newborn and Infants:* In infants, percent body fat can be estimated by a prediction equation derived from four skinfold measures [30]. This method has been validated against DXA in newborns [31] and among children aged 4–10 years [32]. The correlation coefficient of equation-estimated percent body fat in newborns compared to DXA is 0.92 and among children aged 4–10 years, 0.88 [31]. The reliability of these estimates range from 99.5 to 99.8% [30-32]. In the ABC all newborns and infants will have skinfold thickness measured (biceps, triceps, subscapular, and suprailiac) at birth and at each annual visit.

Stage 3: Follow-up after Delivery:

After delivery, mother and child dyad are further followed by e-mail or telephone at 6 weeks and 6 months to collect information on the infants weight and feeding practices, and in a face to face visit at 1, 2, and 3 years after birth. An annual blood sample will be collected from the infant to measure the complete blood count to screen for iron deficiency anemia, and for analysis of glucose, insulin, and lipids. We offer use of a secured study website for participants to enter the baby’s weight, length, and head circumference monthly recorded at their routine well baby visits.

*Assessment of growth and body composition of the infant:* Anthropometric measurements of the child are made annually. Infants are weighed to the nearest 10 g on an electronic scale; length is measured on an infantometer. Head, chest and mid upper arm circumference of the baby are measured to the nearest 0.1 cm using a plastic measuring tape. Skinfold measurements are measured to the nearest 0.2 mm, using skinfold calipers (Holtain, UK) for prediction of body composition. All measures are done by trained personnel, and inter-observer reliability testing is conducted. Crown-heel length which is measured using a length board until 18 months of age, and height will be measured using a Harpenden stadiometer after 18 months of age. Weight is measured with an electronic scale.

*Breastfeeding, Infant diet, and Activity Assessment:* Information on infant feeding practices is collected at 6 weeks and 6 months, and annually by interviewing the mother of the infant/child. Information on initiation of breastfeeding, exclusivity of breastfeeding, duration of breast feeding, and introduction of complementary foods is collected. A validated Infant Feeding Form is used and is a closed ended questionnaire with information about breastfeeding, other feeds and complementary feeds taken during last 7 days [33]. Among mothers who are breastfeeding, breast milk is collected at 6 weeks postpartum. The samples are collected, frozen and stored for future analysis of macronutrient content and environmental toxins such as persistent organic pollutants [34]. The goal is to evaluate the association of breast milk content with adiposity and related metabolic phenotypes. Furthermore, an intervention to promote breastfeeding in the community is being pilot tested as a sub-study. The primary objective of the intervention study is to determine if prenatal training in breastfeeding education of a family member or support person improves the rate of any breastfeeding at 6 weeks post-partum. At age one and 3 years, the mother will complete a 24-hour dietary recall for the child. An activity assessment in the growing child at each annual visit will be performed using a 24-hour activity recall developed and validated for use in young children.

*Infant microbiome:* Stool will be collected from infants at age 1 year. Prior to the annual visit, a stool collection kit will be mailed to families. The kit will contain diaper liners, collection bag and instructions. A diaper liner will be placed in the child’s diaper until stool has been deposited in the liner. The diaper liner with specimen will be placed in the collection bag and refrigerated. The samples will be collected at the annual visit and frozen for future analysis.

*Genetic, Methylation, Gene Expression, Placenta Analysis:* Mother’s and newborn’s DNA is extracted from the buffy coats and used in future genetic association and methylation studies. RNA from leukocytes are extracted from PaxGene tubes, which are collected from newborns at birth. A 1 cm × 1cm biopsy of the placenta is collected from all consenting mothers and stored in RNAlater to enable future placental gene expression and methylation analysis.

*Grandmother’s Interviews:* Grandmothers of Aboriginal ancestry are invited to participate in an individual semi-structured qualitative interview, ensuring that their beliefs are captured using a culturally-sensitive lens. The interview questions are designed to elicit grandmother’s beliefs regarding optimal health behaviours for a woman (1) before pregnancy, (2) during pregnancy, (3) the first 6 weeks postpartum, and (4) optimal behaviours for the family with the new baby in the first year of life. Questions probe their beliefs about diet and feeding practices, sleep, activity, smoking, alcohol, social support, mental health, and intimate partner relationships. The qualitative interviews are analyzed using a constant comparison technique to identify emergent themes, concepts and linkages and used to develop a theory. Grandmother’s beliefs will then be compared to existing evidence-based knowledge and the results of the ABC study and used to inform future education initiatives.

### **Statistical Considerations:**

*Statistical Power:* We estimate that 300 mother-baby dyads will provide high power to address the primary objectives of this study. The primary outcome of the study is newborn adiposity measured by skin fold thickness from 4 locations (triceps, biceps, subscapular, and suprailiac).

#### *Primary Objectives:*

**Objectives 1–3:** For continuous predictors, with 300 newborns we have >80% power to detect an absolute change in percent body fat of 0.82 per 1 SD increase in a given predictor (i.e. maternal weight gain) (two-tailed alpha = 0.05), and we have >90% power to detect an absolute change of 0.96 in percent body fat. For categorical predictors (i.e. maternal gestational diabetes), with 300 newborns we have >80% power to detect an absolute difference in percent body fat of 2.53% when at least 20% have the exposure of interest. There is also sufficient power to detect an absolute difference of 3.24% body fat between top and bottom quartile group extremes (e.g., maternal glucose values or dietary factors). Similar high power is present to test postnatal factors against change in adiposity from birth to age 3 years.

#### Secondary objectives

**Objective 4***Breastfeeding*: Based on available literature amongst Aboriginal women in Canada and input from the lactation consultant from the Six Nations community the usual rate of any breastfeeding in the control group is expected to be 50% at 6 weeks post-delivery. We anticipate our intervention will increase this to 70% and have powered the study to detect a 20% absolute increase.

**Objective 5***Genetics*: We anticipate that the minor allele frequency (MAF) of SNPs of selected candidate genes will be similar to the MAFs we have observed in other cohorts [35]. To study associations of selected candidate SNPs with their respective quantitative traits we anticipate requiring approximately 1,000 babies of Aboriginal origin to have high power. Expansion of ABC to other Aboriginal reservations to achieve this objective is planned. We will also include our 300 participants data in larger genetic consortia of birth cohorts to enable the study of gene-environmental interactions.

**Objective 6***Diet and microbiome*: The infant microbiome diversity at 1 year will be tested for association with infant outcomes at age 3 years. The composition of the infant microbiome will be compared between exposure groups comparing those exposed to maternal gestational diabetes, exclusive breast feeding, and treatment of the infant with antibiotics in the first year of life. The infant microbiome will also be assessed as a potential determinant of child adiposity and insulin resistance at age 3 years. With 300 samples, there will be considerable sample size/power to detect differences in bacterial composition and diversity at infants 1 year of age based on exposure group, compared to prior sample sizes of microbiome studies [36-39].

**Objective 7***Birthweight, adiposity, and ethnicity*: Comparing body fat percentage in Aboriginal newborns to white Caucasian and South Asian newborns in Canada from the FAMILY [40] and START [41] cohorts respectively, we will calculate body fat from the skin fold thickness measures, and after adjusting for differences in gestational age, we will compare the percent body fat per kg of birth weight. Normalizing body composition measurements (e.g. skinfold thickness and body fat) by birth weight has been used as a standard approach to make comparisons across groups of infants of varying body size, including across ethnic categories and sex [42,43]. The estimate of percent body fat in the FAMILY [40] study among white Caucasians (n = 868) is 17% (SD: 4.3%) or 5.1% (1.5%)/kg of birth weight, and it is expected to be 6%/kg birth weight in South Asians. Therefore with 300 Aboriginal newborns, we will have > 80% power to detect an absolute difference in percent body fat/kg birth weight of at least 0.30%, and we have similar high power to detect absolute differences in birth weight as low as 120 grams between Aboriginal and newborns from other ethnic groups. A difference of 0.30%/kg is clinically important as this difference was associated with an increase in insulin resistance of 0.8 units in HOMA in an adolescent cohort, which is equivalent to a 20% increase in risk of incident diabetes after 10 years [44].

**Objective 8***Grandmothers’ study*: Non-probabilistic sampling of 20 to 30 grandmother participants is anticipated, however study enrolment will continue until data saturation is reached. Interviews will be recorded and transcribed verbatim. The text of the interviews will be imported into NVivo-9 software for coding. Data will be analyzed using thematic analysis. Multiple layers of coding will be performed including open, focused, axial and thematic coding. The data will be closely adhered to with sensitivity to emerging subthemes and when saturation is reached, no further interviews will be held. After identification of themes, participants will be invited to have a final interview where the themes will be presented back to them for member-checking and triangulation.

*Statistical Analysis:* Descriptive statistics characterizing maternal and newborn characteristics will be generated. Continuous variables will be reported as means and SD for the normally distributed variables otherwise median and inter-quartile ranges will be reported. Categorical variables will be reported using percentages. Normality of the variables will be examined and appropriate transformations applied if required. All analysis will be considered statistically significant at 5% level. As an example of the multiple sources of data we will accrue from this study a detailed statistical analysis plan for the Aboriginal cohort is provided. We propose to study, the contributions of maternal, newborn, and post-natal factors to the offspring’s adiposity at birth, and the change over time of body fat in the offspring. To do so we will study the effect of multiple types of data (i.e. maternal characteristics such as diet, hypertension, micronutrient status, maternal weight gain, presence of gestational diabetes), infant characteristics (gestational age, feeding type, amount, duration, microbiome profile), certain contextual factors (i.e. socioeconomic status), and in future selected genetic variants (i.e. using a gene score of all known common genetic variants associated with adiposity) on the outcome of adiposity, and change in body fat from birth until 3 years. Our goal will be to identify those determinants and their interactions, which predict change in adiposity as the child grows. A two-staged analytic plan will be used to accomplish this goal. Stage 1: Examining the strongest influences (maternal and newborn characteristics) on adiposity: First, the relationship within each group of determinants (maternal and newborn) and adiposity will be assessed using multi-level growth curve models [45]. Measurement of adiposity over time for the same children will be modeled to show trajectories or slopes (linear or non-linear patterns) as a function of strongly associated and significant influences identified within each factor group (i.e. maternal, infant, genetic). Potential covariates (i.e. contextual factors) will be examined to identify those that are highly correlated with other potential covariates; and only those that have an independent influence will be included. Covariates known to be associated with adiposity within each factor group will be included a priori and then other covariates will be identified using a backward elimination technique. Once a reasonable predictive model has been chosen, all excluded covariates will be added into the model one at a time to identify any missing confounders. We will also apply a stepwise regression approach to assess robustness of our modeling strategy. Previous studies in which models are validated in independent data sets have shown that a fitted regression model is likely to be reliable when the number of independent predictors is less than the total sample size divided by 20 [46].

## Discussion

There is an urgent need to understand maternal and child factors that underlie the early development of adiposity and type 2 diabetes in Aboriginal people. The information generated from this cohort will increase our understanding of the contribution of pre and post natal factors to childhood overweight/obesity and type 2 diabetes, and assist the Six Nations community in developing interventions to prevent early adiposity in Aboriginal children. We anticipate this study will be expanded to include other Aboriginal communities across Canada.

## Competing interests

The authors declare that they have no competing interests.

## Authors’ contributions

GW, JW, RM, RA, SM, KKM, KKT, SSA made substantial contributions to conception and design of the study and participated in the writing of the manuscript. All authors read and approved the final manuscript.

## Pre-publication history

The pre-publication history for this paper can be accessed here:

http://www.biomedcentral.com/1471-2458/13/608/prepub

## Investigators

The Principal Investigator (PI) of this proposal is **Dr. Sonia Anand**, (Professor of Medicine and Epidemiology). The Deputy PI is **Dr. Gita Wahi,** (Assistant Professor of Pediatrics) McMaster University. **Co-Investigators** from McMaster include: **Dr. Koon Teo**, (Medicine, Epidemiology); **Dr. Katherine Morrison**, (Pediatrics); **Dr. Sarah McDonald,** (Obstetrics and Gynecology); **Dr. Joseph Beyene**, (Biostatistics); **Dr. David Meyre,** (Genetic Epidemiology), **Dr. Alison Holloway** (Obstetrics and Gynecology); **Dr. Guillaume Pare,** (Genetics), **Dr. Rebecca Anglin,** (Psychiatry and Medicine); **Co-Investigators** from Six Nations include: **Ruby Miller,** Director of Health Services at Six Nations**, Julie Wilson** Director, Six Nations Birthing Centre. **Other co-investigators include Dr. Zohra Docrat** (Brantford General Hospital) **Dr. Ravi Retnakaran**, (University of Toronto, Medicine).

## Study team

Bonnie Davis, Dipika Desai, Phyllis Hill, Trista Hill, Laurie Jacobs, Sujane Kandasamy, Stephanie McDonald, Kristi Shawana, Sharon Smoke.
